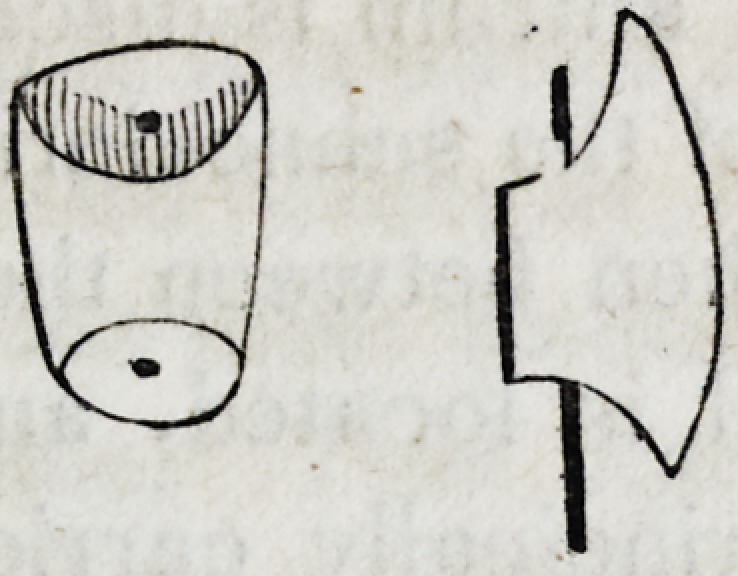# A Treatise on Mechanical Dentistry

**Published:** 1842-06

**Authors:** Solyman Brown


THE AMERICAN

### JOURNAL AND LIBRARY

OF

### Dental Science.

Vol. II.] JUNE, 1842. [No. 4.

### ARTICLE I.

*A Treatise on Mechanical Dentistry.* By Solyman Brown, M. D., D. D. S.

(Concluded from page 247.)

### CHAPTER III.

### *Of the insertion of entire sets of artificial teeth, both upper and lower, on plates of gold, without clasps or springs.*

61. It often happens, in this age of luxurious refinement, effeminate ease, and unnatural alimentation, that persons of both sexes, and of all classes of society, lose successively every tooth in both jaws; but more frequently in the upper maxillary arch. The dental operator who understands his business, although he deplores the folly or misfortune of his patient, is never more confident of ultimate success than in treating cases of this description, when he is permitted to exercise his best judgment in the employment of his utmost skill on the most suitable materials. As there is no longer a necessity of attaching his work to other teeth, which are liable to be soon removed either by decay or dislocations, he can construct an entire set of teeth, set upon a strong plate of gold, which shall fulfil all the reasonable expectations of the patient in regard to appearance, speech and mastication, and which shall moreover be so durable in its character as to render the annual expense of small amount for many years to come.

62. To secure entire success in the fabrication of these entire sets of artificial teeth, the utmost care must be taken to remove all the roots of the natural teeth, and to bring the gums and adjacent parts into a perfectly healthy condition. In ordinary cases, time alone, without any sanatary remedies, will effect this object, provided a sufficient period be allowed after the removal of the natural roots. Not only must the cavities which the roots occupied be wholly filled up, but the alveolar processes must be thoroughly absorbed, so that no material changes shall occur after the wax impression shall have been taken, from which the working models are to be made.

63. It is impossible to prescribe definite periods for all the cases that may occur in a promiscuous practice, during which this healing process, together with the absorption of the bone, will be perfected. Much depends on the age, habits and health of the patient, and not a little on the circumstances under which the natural teeth are shed. In some subjects, the alveoli are nearly or wholly absorbed, before the roots of the natural teeth lose their hold upon the integuments: in others, several sound teeth are extracted, around which the alveoli were perfectly preserved. From all these causes it must be evident, that each distinct case, possesses an idiosyncrasy of character peculiar to itself, and must be treated accordingly. It is proper to say here, that a few weeks will be sufficient for the preparation of some mouths for taking an impression; whereas, months will be required in a majority of instances.

64. When the operator becomes convinced that the gums are well healed and settled, and that no further changes are to be feared, which will endanger the permanent and complete success of the operation, he should procure very ample metallic cases to contain his wax, so that the impression may extend to every part of the maxillary arch on which the plate must rest; taking care that the wax shall reach the extremity of either jaw towards the angle of articulation, and spread itself over at least two-thirds of the palatial concavity, when the upper teeth are to be restored, and moreover, that the wax come foward on the labial side of the arch as far as the muscles and integuments will permit. In taking these large impressions, let the wax be pure and without any admixture of olive oil, and just soft enough to receive the impression without too great pressure on the parts, and yet not so soft as to be injured while withdrawing it from the mouth. The proper consistency of the wax and the necessary shape and strength of the metal case, which contains it, are of great moment in the attempt to secure a satisfactory result; but in this case as in many others, experience is the only competent instructor.

65. Inasmuch as the pieces which I am now describing, are those which by some have been called "suction plates," and by others are said to be retained in position by *atmospheric pressure,* I deem it proper in this place, to express an opinion on the subject, resulting not only from conversation with distinguished dentists, but from years of personal experience and observation, during which I have had occasion to inspect pieces of this description constructed by some of the most distinguished artists of both hemispheres.

66. One result of my inquiry and observation has been, that few pieces of this kind are fixed firmly upon the gum, and used successfully for purposes of mastication, by mere suction, or atmospheric pressure, without any aid from the tongue, lips, cheeks, and antagonizing jaw. There is indeed, in favourable cases, a very firm adhesion of the plate to the subjacent parts after it has been some time worn, and thus closely fitted to the gum and palate. But this adhesion which is that of two well adapted surfaces, from between which the atmosphere is excluded either by impact, or by liquids, or both combined, could never be effected unless the plate were held firmly in its place, for a season, either by the bold relief of the alveolar projection, or by the lips, tongue, teeth and opposing jaw; and in fact, these surrounding parts—together with the contained convexity of the projecting gum, contribute not merely *partially,* but *mainly* to the preservation of the piece in its just position. The manner in which the surrounding parts operate to secure such a plate *in situ,* are too well know to need any illustration even to the youngest student of our art; but the manner in which the bold *bas relief* of a prominent alveolar projection, may and often does operate to sustain a plate, from beneath which the atmosphere has not been excluded, shall be explained by a simple diagram.

Let *A B* in the above figure, be a cylinder of wood, metal or any other substance, not more yielding than the alveolar ridge of the human mouth when denuded of its teeth; and let *C D* be a plate of metal so bent as to embrace the cylinder firmly in the parts adjacent to its two straight sides. It is very manifest, that such a plate might be sustained very firmly in its connexion with the cylinder, even though a stratum of air of any supposable thickness intervened between the cylinder and plate.

It is indeed quite certain, that the firmness of this adhesion would be much increased if the cylinder were so fitted to the plate, as that the air were wholly excluded, and this only proves what I wish to impress strongly on the mind of the dental student, that even when all other circumstances are most favorable to the success of his operation, the perfect adaptation of plates like this to the subjacent parts, is of the utmost importance. The fact that a *perfect fit*, as it is called, excludes the particles of food, and also renders the plate more tolerable to the tongue, would of itself impel every neat and finished operator, to give to his plate the best possible adaptation to the parts beneath.

67. Another fact, with which observation has furnished me, is, that inasmuch as the lips and cheeks contribute greatly to the stability of the position of an entire set; the artificial teeth should extend as far back as possible on the plate, in order to present as large a surface as convenient to the sustaining action of the muscles of the cheek. For a similar reason, the portion of the gold plate resting upon the palate directly over the tongue, in what is vulgarly called the roof of the mouth, should be as broad as circumstances will permit, in order that the superior surface of the tongue may lend its important aid, when required, in sustaining the artificial fixture in its place.

68. Experience and observation teach moreover, that the gold plate must pass upwards as high as the muscles will permit, on the anterior and outer surface of the gum, between the cheeks and the maxillary arch. This not only increases the surface, thereby augmenting the force of adhesion, but it assists in preventing the plate from moving either laterally or backwards, in the act of mastication.

69. A proper thickness of the gold plate is matter of primary consideration, in these entire sets of teeth; and the thickness must vary in the direct ratio of the flatness or prominence of the alveolar ridge. The rule invariably is, that the plate shall be as light as is compatible with the strength required. Very flat gums require thicker plates than those which are more convex and protuberant, on the simple principle that a flat ribbon of metal of any given breadth and thickness, will bend in the direction of its length more readily than the same plate bent laterally into the semi-segment of a hollow cylinder.

If *A* represent a flat metallic plate of any length and breadth, and *B* a cylindrical plate of equal dimensions, the strength of the latter will be many times greater than that of the former, in proportion to the convexity of its curvature. Hence it follows that the deeper the convexity of any gold plate for the mouth, and the broader its superficial area, the thinner may be the plate. And on the other hand, when the plate is narrow and flat, it must be increased in thickness until it shall possess sufficient strength to resist the ordinary forces to which it must be necessarily subjected.

70. Since, as we have already said, every plate worn in the mouth should be as light as the strength demanded will permit, we have another among many solid reasons for preferring gold to any other metal for setting artificial teeth. Platina would resist the action of corrosive agents better than gold, but a much greater weight and bulk of this metal would be required, to give the strength of a similar gold plate.

71. The following is a drawing from a cast of one of the most prominent gums, to which I have been called upon to adjust a suction plate during the period of my professional practice.

The depression at *A* below the prominence at *B C D* is full three-fourths of an inch. To a mouth like this there can be no difficulty in adjusting a suction plate with the most perfect success. If the model be correctly taken, the plate rather thin, and proportionally broad, it will require no more than ordinary mechanical skill to construct a piece which will remain firmly in its place, even in the act of masticating the hardest kinds of food.

72. But the ability of such a piece, to sustain the office of an implement of mastication, depends greatly on the position of the teeth which are set upon it, and on the manner in which they meet those of the lower jaw. If the front incisors should be set too far forward on the plate, and if there should be no molar teeth in the lower jaw to countervail their action on the lower incisors, it will be manifest that the whole will be thrown forward, in closing the mouth.

In the cut here given, if we suppose the upper incisors at *A*, to strike wholly over and in front of the lower incisors at *C*, inasmuch as there is nothing to sustain the point of the plate at *B*, it will inevitably fall away from the gum, whereupon the whole will be projected forward whenever the mouth shall close, and render the piece useless. Such an arrangement could be made available only by supplying the deficiencies in the lower jaw. If this is not done, the front teeth of the artificial piece must be removed backward until they strike perpendicularly upon the lower incisors, in which case the centre of gravity of the artificial piece will in most instances, be so supported as to render the work available for all the important uses to which the teeth are usually applied.

**73.** After the dental operator has formed his plan as to the position which he wishes the teeth to assume on the plate, and also as to the manner in which he intends the substitutes shall meet the natural teeth of the lower jaw, the best method of proceeding is as follows: After striking the plate as heretofore described, and after fitting the gold backs to the teeth which have been selected for the purpose, cover the outer surface of the plate which may be called the *lingual* surface, with softened beeswax, equal in thickness to the length of the teeth, and adjust the teeth as nearly as possible in the required position, taking care to grind each tooth down to the plate with the greatest accuracy, in such manner that the gold back of each tooth shall touch the plate. There are some advantages, in certain cases, in using sealing-wax instead of beeswax, in thus temporarily attaching the teeth to the plate. When this is used the flame of a lamp or candle may be employed to melt the wax as it shall be needed for each tooth.

**74.** In this stage of the progress, try the whole into the mouth of the patient and correct the position of the several teeth so that in all respects they shall stand as desired. This process of adjusting will be readily effected, while the wax is preserved by heat in a softened state. When all the teeth stand exactly as all parties desire, remove the whole from the mouth carefully, and encompass all the teeth, together with the plate, with plaster of paris, leaving the wax uncovered. When the plaster shall become thoroughly hard, immerse the whole in warm water, in order to soften the wax, or cut it away with a penknife, leaving the teeth and plate firmly imbedded in the plaster. In this as well as other cases where the plaster must be subjected to the action of the blow-pipe, sand should be mixed with the plaster to prevent its cracking, or if this precaution should have been neglected, the plaster may be encompassed with a few turns of fine iron wire, as already directed.

75. After the gold plate and backs of the teeth have been divested of the wax, they should be well washed in clean water, when the piece will be ready for soldering. In large pieces like this, where great strength and durability are required, the utmost care must be used to employ solder enough not only to render the work beautiful, but to give the stability required; for, as I have already remarked, a good piece of work never gives way at the junction of the plate with the backs of the teeth, inasmuch as that is by far the strongest part of a good piece. In soldering large plates of this sort, the flame of the blow-pipe should be applied cautiously at first, and afterwards augmented gradually till the whole mass is heated to redness; after which a concentration of the jet of flame should be brought to bear upon the back of each of the teeth successively till the solder has all assumed the desired form and position, throughout the entire range. After cooling, cleaning, and polishing, the piece will be ready for insertion.

76. As the cleaning and polishing of such large plates, is an important operation as regards their beauty, I will recapitulate the process, and describe it a little more minutely than I have yet done. When the piece has been boiled for a few seconds in diluted sulphuric acid, or suffered to lie a few minutes in diluted muriatic acid, without boiling, let the edge of which rests on the palatial arch be a little bent upwards by the use of a small riveting hammer, while the plate rests on the end of a steel rod half an inch in diameter, rounded at the end in the form of a hemisphere.

The shape of the rod, and the form and size of the hammer, are represented in the following wood-cut.

The sharp and broad edge of the hammer must be applied with repeated strokes and a skilful hand to the lingual side of the inner edge of the plate until it shall effect the object to the extent desired. There is but one method of avoiding the necessity of this operation, which is to pare away the plaster cast upon which the metal casts are to be made, along the inner edge of the plate, so that the plate in being struck between the metal casts, shall be properly bent upwards without the use of the hammer afterwards. This trimming of the cast, however, will be more difficult than the method first described, especially to the inexperienced.

77. Let the plate in the next place be filed very smooth, in all parts, commencing with coarser gold-files and finishing with very fine ones. In the next place, rub the plate smooth with a silversmith's polishing stone, which may be cut with a fine saw into any shape desired. After this, use rotten-stone and sweet oil, applied with leather attached to the end of a piece of soft wood; afterwards apply dry Spanish white, crocus, or rouge, and finish by washing in clean water. To those portions of the plate which cannot be reached with the leather, the above-named substances may be applied on threads of silk, flax, hemp or cotton. When threads are thus used they may be fastened at both ends to some fixed points in the manner of the horse-hair of the bow of a violin, and motion may be given to the piece along these filaments to which the polishing substances have been previously applied. When the whole is finished and ready to be inserted in the mouth, it presents the following appearance.

78. It sometimes happens that the maxillary ridge is so flat, that the action of the lips, cheeks and tongue, together with the slight degree of adhesion which takes place when the plate is first inserted, are incapable without a little practice of keeping the piece in its place, whereupon being left to the action of gravitation, it falls whenever the mouth is opened. To remedy this difficulty which is removed by a little experience, and which is felt less and less as the gum becomes adapted to the plate, it is sometimes necessary to wear a piece of soft leather, either that of the chamois goat, or well-dressed lambskin, cut to the exact size of the gold-plate, and moistened with water. By this means the adhesion is promoted to such a degree, that the piece may be worn with little comparative difficulty. It is often true, in the case of aged persons, that the alveolar processes become so completely absorbed, and the parts upon which the plate must rest, so nearly flat, that the difficulty of wearing a suction plate is greatly increased. In such cases the use of springs must be adopted, such as will be hereafter described, or the patient must dispense with artificial teeth altogether.

79. As the foregoing description of a suction plate, is applicable only to the upper jaw, inasmuch as the principle of cohesive attraction or atmospheric pressure, does not apply to any great extent, to plates worn upon the narrow alveolar ridge of the lower jaw, it may be proper here to remark, that the attraction of gravitation necessarily operates in some degree to counterbalance this defect in the lower plates. These pieces will of course remain in their places by gravitation alone, provided the food, the tongue, the lips, and the cheeks do not displace them. But all these causes are so apt to render lower plates useless without some artificial means to keep them in position, that I shall deem it necessary to employ springs in most instances; but as there are a few cases in which under pieces can be worn without springs, I shall present a drawing of one of them as follows :

80. In these plates for the under jaw, the whole process of construction, will be similar to that already described, with the difference, that as the edges of the plate must approach as near as possible to the muscles on either side of the alveolar ridge, without wounding them, and as the plate at least can be but narrow, it become imperative that it should be much thicker than plates for the upper jaw. This increased thickness will both impart the required strength, and present a less trenchant edge to the muscles and integuments that necessarily move upon it. There is an excellent method of removing this difficulty of the sharp edges of the lower plate, when the condition of the mouth will admit of it. In many cases the absorption of the parts becomes so considerable, as to leave sufficient room in the mouth, to construct the lower plate *double*, especially that portion of it which pertains to the back part of the mouth, sustaining the molar teeth. The following method of procedure will enable the operator to construct a double plate with facility. After the ordinary plate has been completed agreeably to directions already given, cover the upper or lingual surface with beeswax, and mould or cut it into such form as shall represent the exact position which it is desired that the upper plate shall assume. Lay the whole on the tin-cast upon which the plate was struck, and after oiling the surfaces of the wax, plate and mould, with olive oil, cover the whole with plaster, sufficiently thick to give it the required strength. This will form the basis of a new set of metallic castings, upon which a second plate may be constructed, which being soldered to the first plate, and carefully filed and polished, will present very smooth and cylindrical surfaces to the tongue, cheeks and lips. This sort of double plate, which has long been familiar to the profession, is quite unlike those patented machines of modern invention, provided with flute-holes like the lamprey, for the storage of all kinds of filth, both solid and fluid, which can be collected from the mouth, fomented by heat, and rendered execrable by fermentation. But as these portable nuisances have gone, for the benefit of posterity, to the tomb of the Capulets, I have resuscitated their memory merely to say, that those double plates which I here recommend, in certain cases, are wholly unlike them, inasmuch as they are merely air vessels, and not depositories of garbage. One advantage of these double plates for the lower jaw, in addition to that already mentioned, is that they enable the artist to use teeth of uniform length, giving greater symmetry and beauty to the work—and besides this the strength of the piece will be augmented many fold. It should be moreover known to the profession generally, that the market is now supplied with *gum-teeth*, as they are technically called, of great beauty, which are very useful in those cases of considerable alveolar absorption, where double plates are not employed.

81. Before entering upon the subject of double sets with springs, which will engage our attention in the next chapter, it may perhaps, be useful to the student to say a few words in relation to the several kinds of mineral teeth which have been offered to the profession from time to time by the principal manufacturers of Europe and America. Until a few years ago the French dentists took the lead in this branch of manufacture, and supplied not only their own country, but foreign markets. If we are to judge of the French teeth from the specimens sent to this country while there was a demand for them, it must be confessed that although the material of both the body and the enamel, was exceedingly well calculated to resist the action of the blow-pipe, and although they were in some instances well coloured, yet as to beauty of form and the method of attachment to metallic plates, they were vastly inferior to those of American fabrication which have now wholly supplanted them. And in relation to two of these points of superiority, I am compelled to acknowledge that I have never yet seen any artificial mineral teeth, so elegantly formed, and colored so perfectly to nature, as those manufactured by Mr. C. Ash, of London, who has kindly forwarded to me a few sets to be exhibited next summer at the meeting of the American Society of Dental Surgeons in Boston. But, as these teeth manufactured by Mr. Ash do not resist the action of the blow-pipe in such a manner as to be capable of being soldered to plates with solder of sufficient firmness to resist the action of the fluids of the mouth, I am confident that some of our American dentists, among whom I am happy to rank Mr. Samuel W. Stockton, of Philadelphia, and Mr. James Alcock, of New York, have presented to the profession the best specimens of incorruptible teeth, of which we have any account in the history of our art. And yet when I contemplate the undeniable superiority of the teeth fabricated by Mr. Ash, of London, as regards beauty of form and perfection of coloring, I cannot allow myself to doubt that higher points of perfection are still to be attained in the production of this species of manufacture, as regards all those particulars in which the teeth now in market are found deficient. There is scope enough to call into activity the energies of the most exalted genius, and a field in which enterprise and perseverance will reap a harvest of gold, when the profession confess that they are wholly satisfied with the quality of this article now so essential to the perfection of our art. But I should esteem myself chargeable with manifest injustice and ingratitude towards those who have done so much for the improvement of mineral teeth, for having spoken thus of the defects of the present fabrics, did I not also express my joy and astonishment that so much has been done already during the present century for this manufacture.

82. Of the various modes of constructing what are technically called *plate teeth*, I shall deem it necessary to introduce but three on this occasion, as being the most conspicuous among the many kinds in vogue.

The French plate teeth so long as they continued to come to our market, were constructed as follows:

Each tooth had a longitudinal groove on its posterior surface, with three points or pivots of platina set firmly in the body of the tooth during its fabrication, to which a gold or platina wire might be soldered. To this wire when ground to the level of the surface of the tooth, a metal back was to be attached by soldering. Teeth thus constructed possess a good degree of strength, but are not easily replaced in case of accident, and therefore the dentists of the United States adopted the following improvement.

Two small platina pivots of wire, are inserted firmly in the body of the tooth during the manufacture, designed to be inserted into two corresponding holes in the metal plate. These pivots can be both rivetted or soldered to the metal backs. Dr. Harrington, of Philadelphia, has recently secured a patent for an improvement in these pivots, which consists in forming a head on that end of the pivot which is inserted into the tooth, as well to prevent it from being drawn out, as to enable the tooth to sustain the strokes of the rivetting hammer. In the fabrication of the American teeth just described, many individuals of eminent mechanical genius, have been for some years ardently engaged. Many of these dentists manufacture only for their own use, and succeed in producing teeth of good quality. Among those who have manufactured for the market, Mr. S. W. Stockton, of Philadelphia, has been most generally patronized, and has received more frequently than any other individual, the medals and diplomas of the American Institute. Mr. James Alcock, of New York, has also succeeded, after years of persevering experiment, in fabricating beautiful teeth, and has likewise obtained the medal of the Institute.

83. The only remaining kind of mineral teeth of which I propose to speak, is that from the manufactory of Mr. C. Ash, of

London. His teeth differ from those already described, in having a central, longitudinal cavity or orifice, bushed with gold, for the reception of a pivot of the same metal, as follows:

One of the advantages of this method of construction, is that teeth of this kind are adapted as well for insertion on roots as on plates of metal. Their great excellency is their surpassing beauty of form and colour; and when these properties can be made compatible with a material capable of resisting the action of the blow-pipe, little will be left to be resolved by future generations in the problem of constructing artificial teeth. But the period when this desirable object will be attained, is left to conjecture, and I deem it not too much to say that, the fortunate individual who successfully resolves this difficult problem and unites the separate excellencies of those three kinds of teeth, will not only insure to himself and his family an ample fortune, provided he conduct the exclusive manufacture with spirit and skill, but will deserve the general thanks of the profession and the gratitude of mankind.

[TO BE CONTINUED.]

## Figures and Tables

**Figure f1:**
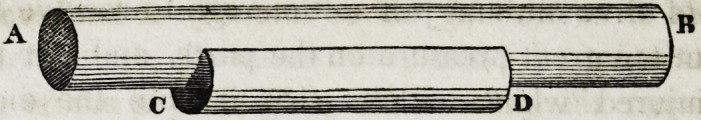


**Figure f2:**
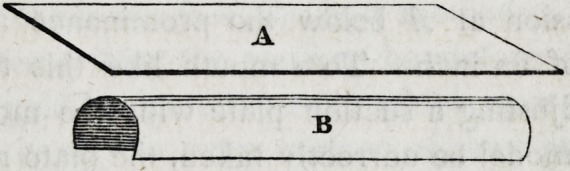


**Figure f3:**
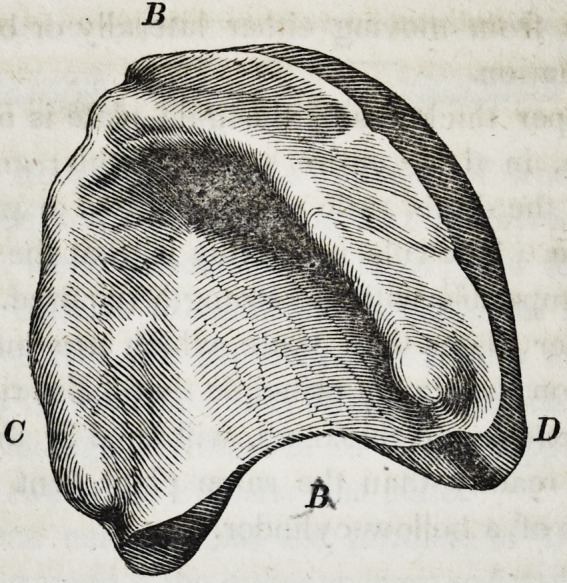


**Figure f4:**
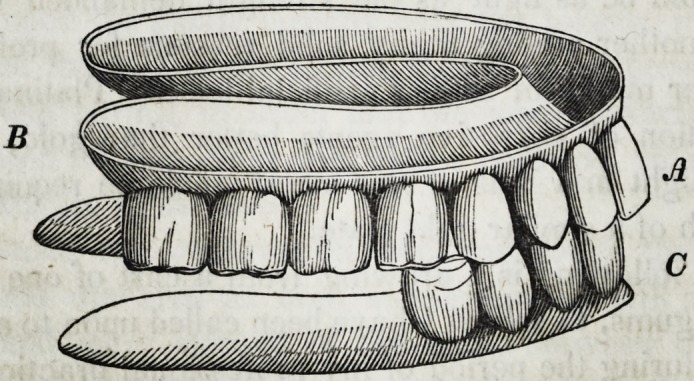


**Figure f5:**
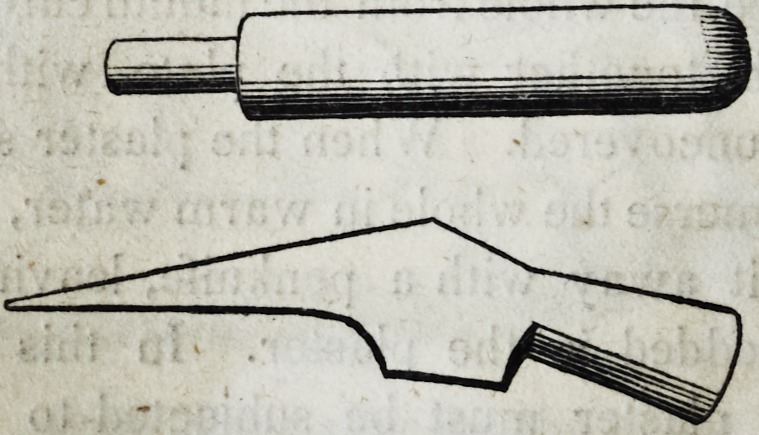


**Figure f6:**
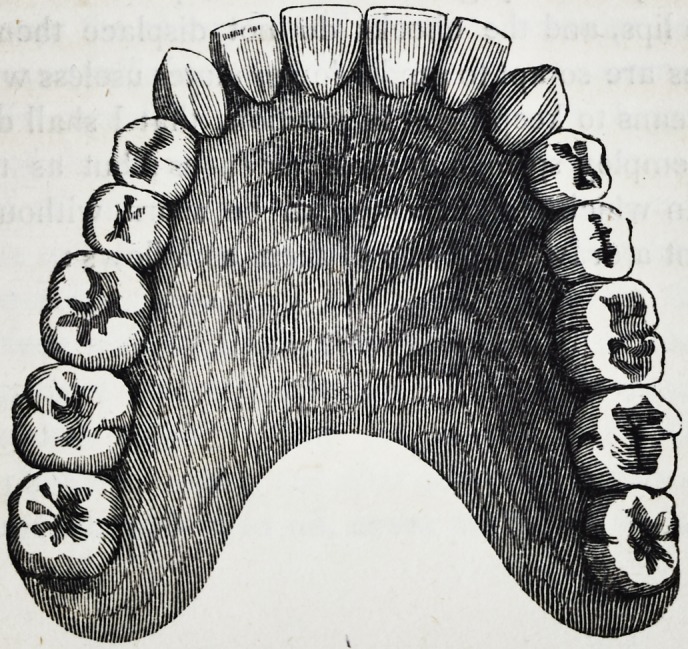


**Figure f7:**
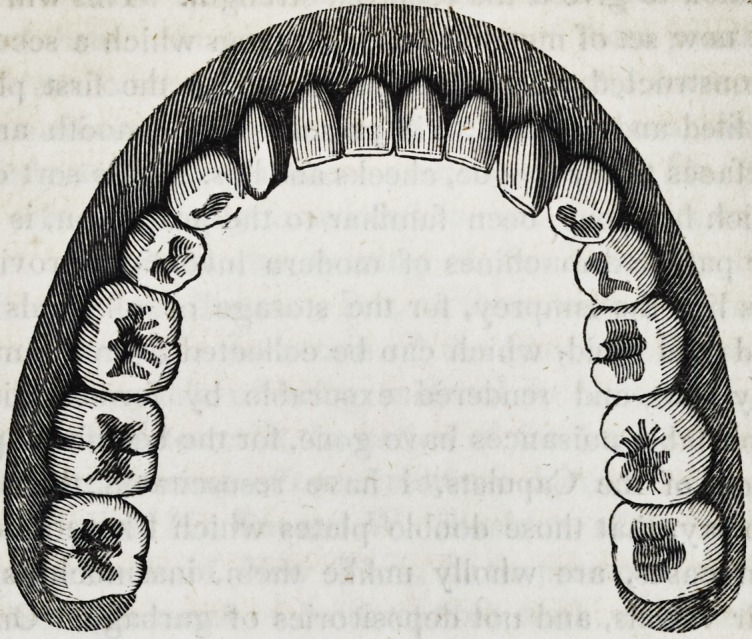


**Figure f8:**
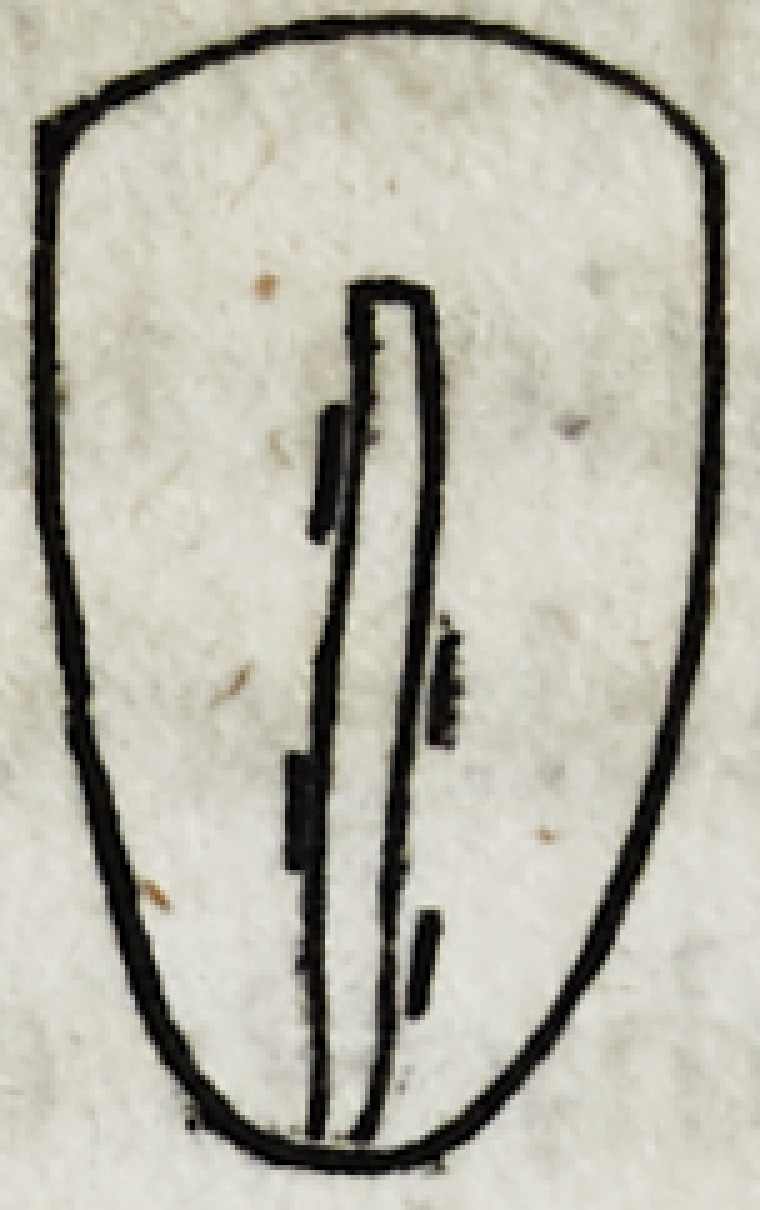


**Figure f9:**
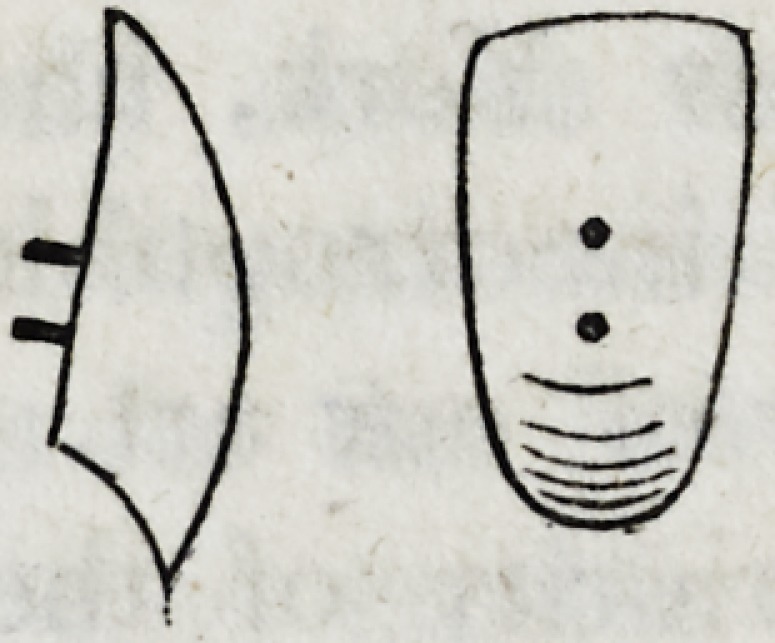


**Figure f10:**